# A Rare Third Ventricular Dermoid Cyst in an Adult With Imaging Characteristics Consistent With a Colloid Cyst

**DOI:** 10.7759/cureus.21172

**Published:** 2022-01-12

**Authors:** Cody Woodhouse, Khaled Abdel Aziz, Dorian M Kusyk, Kristen Stabingas, Feifan Chen, Jonathan Pace, Jody Leonardo

**Affiliations:** 1 Department of Neurosurgery, Allegheny Health Network, Pittsburgh, USA; 2 Department of Neurosurgery, Lake Erie College of Osteopathic Medicine (LECOM), Erie, USA; 3 Department of Neurological Surgery, Allegheny Health Network, Pittsburgh, USA; 4 Department of Pathology, Allegheny Health Network, Pittsburgh, USA

**Keywords:** cranial pathology, hydrocephalus, intraventricular hemorrhage, colloid cyst, dermoid cyst

## Abstract

A 64-year-old male presented with spontaneous intracerebral hemorrhage and obstructive hydrocephalus without evidence of a third ventricular mass in 2019. The patient was lost to follow-up and re-admitted one year later for hydrocephalus secondary to a third ventricular mass. Imaging characteristics were consistent with a colloid cyst, which was the presumptive diagnosis. A transcallosal transchoroidal approach was utilized for cyst resection. The cyst wall was carefully incised, releasing flakey, partially solid contents which were grossly inconsistent with a colloid cyst. Due to the concern of iatrogenic cyst rupture in the setting of unknown diagnosis, the patient was placed on steroids post-operatively. Surgical specimens sent at the time of surgery were consistent with dermoid cyst. We present the first reported case of a third ventricular dermoid cyst in an adult initially misdiagnosed as a colloid cyst based on imaging characteristics.

## Introduction

Dermoid cysts are benign lesions that represent around 0.04-0.7% of intracranial tumors [1,2]. Most are thought to be congenital, arising between the third and fifth week of gestation when the surface ectoderm fails to separate completely from the underlying neural tube [3]. Congenital dermoid cysts are most commonly located at midline skull base regions [2]. Alternatively, they can arise at any age when there is traumatic implantation of skin elements into underlying tissues such as a lumbar puncture [3].

When found within the ventricular system, dermoid cysts are typically located in the fourth ventricle [4,5]. There have been only two reported third ventricular dermoid cysts in children, while none have been reported in adults [6,7]. In contrast, the most common third ventricular tumors are colloid cysts [8]. Despite variable radiologic characteristics, colloid cysts often top the differential diagnoses for round third ventricular tumors. We present the first reported case of a third ventricular dermoid cyst in an adult initially misdiagnosed as a colloid cyst along with a review of related cases in the literature [6,7].

## Case presentation

**Figure 1:**
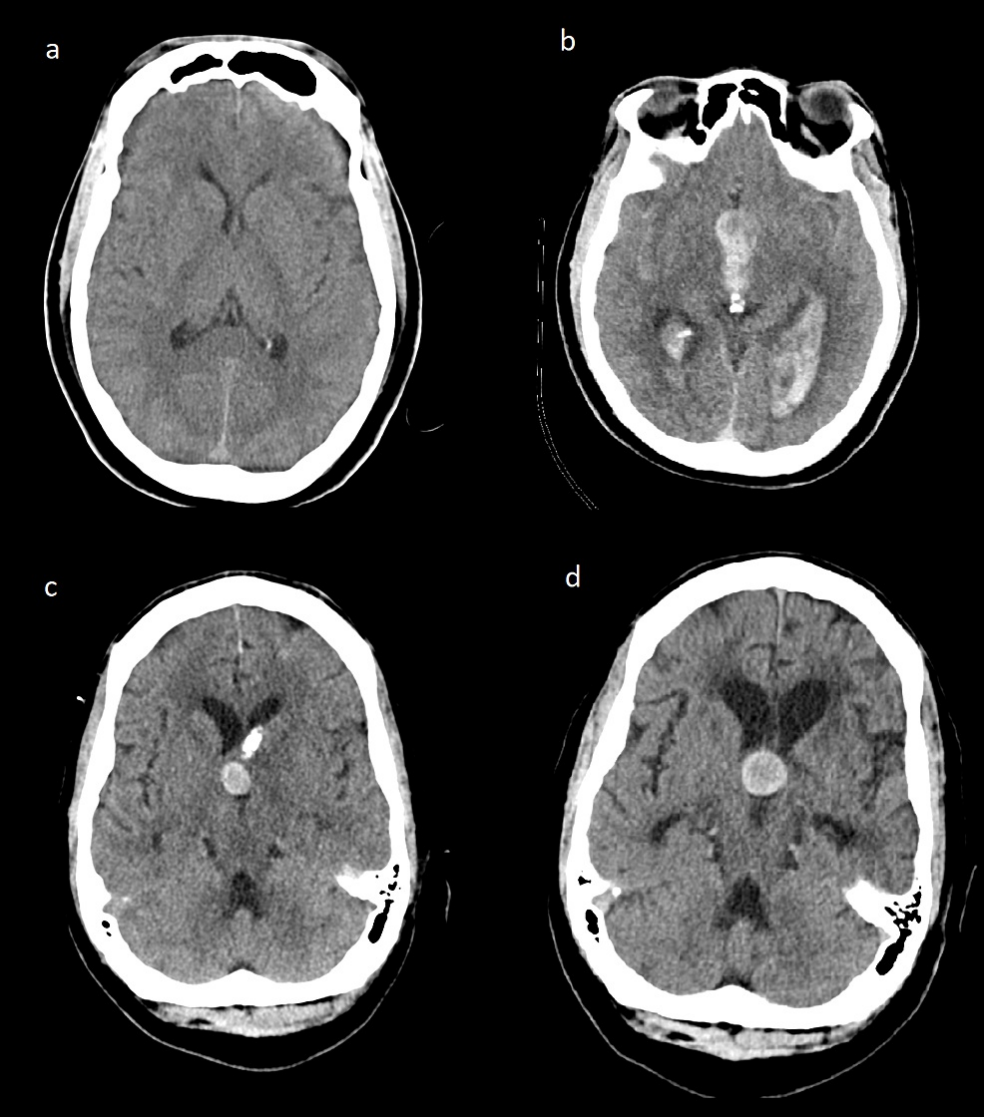
CT scan of the patient over 12 years of period CT head (a) May 27, 2008, without any intracranial abnormalities; (b) July 24, 2019, demonstrating intraventricular hemorrhage and hydrocephalus; (c) August 14, 2019, demonstrating resolution of IVH with evidence of third ventricular mass adjacent to the foramen of Monro and external ventricular drain; (d) November 25, 2020, demonstrating obstructive hydrocephalus secondary to a hyperdense third ventricular mass. IVH: intraventricular hemorrhage

In July of 2019, a 64-year-old male presented with non-ST elevation myocardial infarction (NSTEMI) who underwent cardiac catheterization. Shortly after the procedure, he had altered mental status with additional symptoms of nausea and emesis. A comparative computed tomography scan of the head (CTH) from 2008 demonstrated no abnormal findings and no evidence of a third ventricular mass (Figure 1a). CTH from this admission also demonstrated no sign of underlying intraparenchymal or third ventricular mass (Figure 1b). The patient's hydrocephalus was urgently managed with an external ventricular drain (EVD). Prior to removal of the EVD, repeated CT head demonstrated resolving intracerebral hemorrhage (ICH) with residual intraventricular hemorrhage (IVH). There was no mention in the radiology report of an underlying third ventricular lesion; however, upon review of the imaging, there appears to be evidence of a spherical mass at the foramen of Monro (Figure 1c). The EVD was weaned and patient was discharged to a rehabilitation facility shortly after. The patient was lost to follow-up. The patient was readmitted in November 2020 after experiencing altered mental status for several days. He was found to be in diabetic ketoacidosis (DKA) and a CTH demonstrated obstructive hydrocephalus secondary to a round, hyperdense third ventricular mass (Figure 1d).

**Figure 2:**
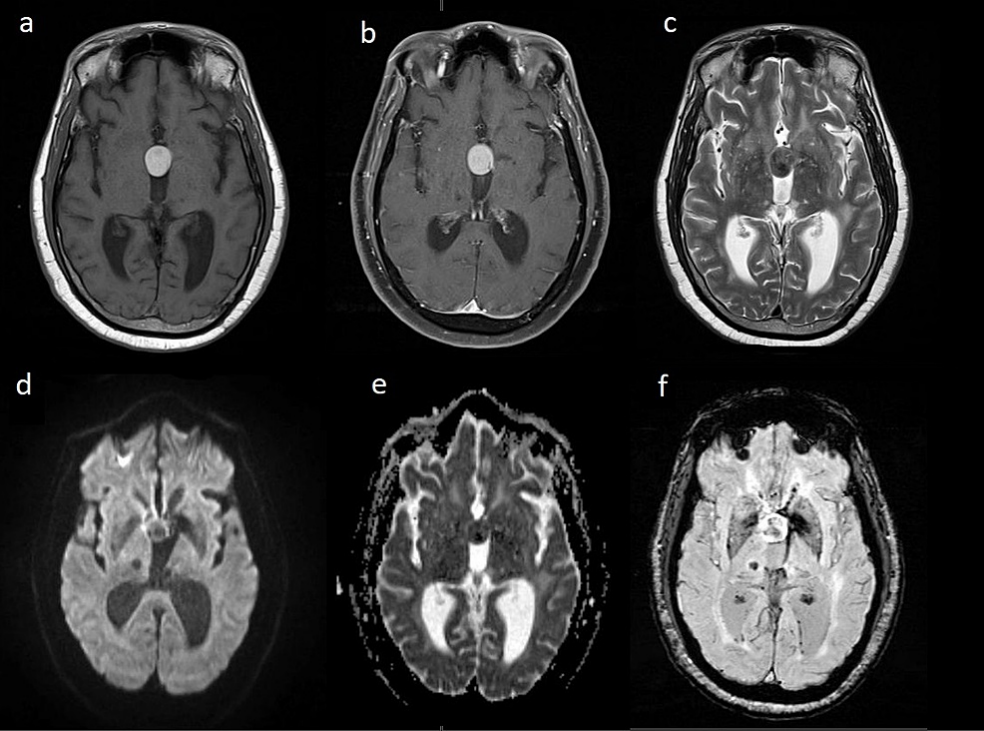
MRI brain, November 27, 2020, further characterizing third ventricular mass (a) T1-weighted image showing hyperintense third ventricular lesion; (b) T1 contrast-enhanced image showing hyperintense third ventricular lesion; (c) T2-weighted image showing heterogeneously hypointense third ventricular lesions; (d) DWI demonstrating no diffusion restriction within the third ventricular lesion; (e) no ADC correlate within the third ventricular lesion; (f) SWI demonstrating no hemorrhage or calcifications within the third ventricular lesion. DWI: diffusion-weighted imaging; ADC: apparent diffusion coefficient; SWI: susceptibility-weighted imaging

An EVD was placed, and the patient was started on a continuous insulin infusion for management of the DKA. An MRI was obtained which demonstrated a third ventricular lesion that was T1 hyperintense, showed homogenous contrast enhancement with an area of hypointensity, and was T2 heterogeneously hypointense without diffusion restriction (Figures 2a-2f).

The patient underwent a right-sided interhemispheric transcallosal transchoroidal approach for removal of the presumed colloid cyst. The cyst wall was carefully incised, releasing flakey, partially solid contents which were inconsistent with the presumed imaging diagnosis of the colloid cyst. Multiple intra-operative specimens were sent to the pathology department for frozen and permanent preparation. The cyst was debulked. The ventricular system was carefully irrigated with meticulous attention in an effort to evacuate residual cyst components. At the conclusion of the case, the pathologist was unable to make a preliminary diagnosis, citing the need for further permanent preparation stains and analysis. Due to the concern of iatrogenic cyst rupture in the setting of unknown diagnosis, the patient was placed on steroids post-operatively.

**Figure 3:**
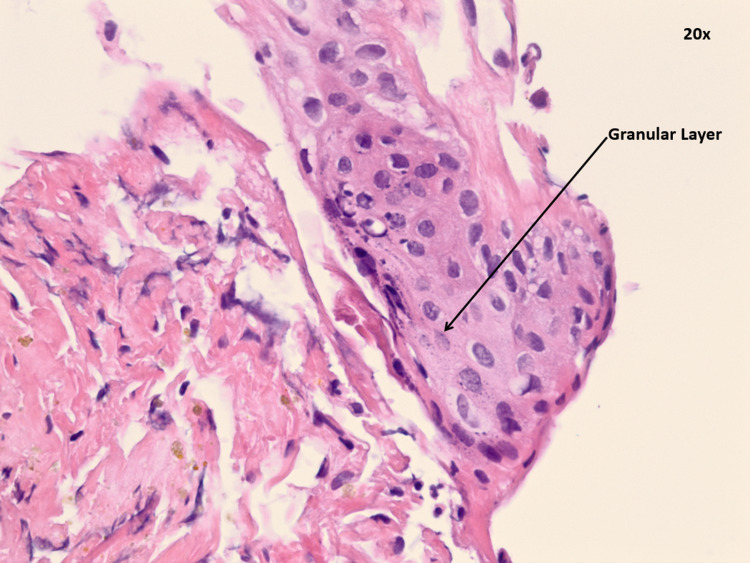
Histology of third ventricular lesion consistent with dermoid cyst

Based on tissue analysis, the lesion’s features were consistent with those of a dermoid cyst. It contained keratin debris, stratified squamous lining, acute inflammatory cells, and a focal area with a granular layer (Figure 3). There were no malignant cells. Immediate post-operative course was unremarkable and he was discharged to a nursing home.

## Discussion

Dermoid tumors are rare and benign lesions that usually occur along the midline. They rarely occur within the ventricular system and when they do, they are typically found in the fourth ventricle [9]. They are usually sporadic, but half are associated with other congenital anomalies such as dermal sinus tracts [5,9]. The mean age of presentation of patients with intracranial dermoid cysts is around 15 years, though patients may present in the third or fourth decade of life as well [5].

Dermoid cysts typically appear as non-enhancing, hypodense lesions on CT secondary to fat generated by sebaceous secretions [2,3,5]. Rarely, dermoid cysts can mimic hemorrhage and appear hyperdense on CT [5,10]. On MRI, they are T1 hyperintense and have a variable T2 signal [2,5]. In contrast, a colloid cyst is typically a hyperdense, well-defined round or oval mass on CT imaging [8,11,12]. On MRI, they typically appear hyperintense on T1 and hypointense on T2 [8,12]. Although these are considered typical findings, there is a high rate of variability in their radiologic characteristics. Given their variability, colloid cysts are often the first on the differential diagnosis for third ventricular mass lesions. Though our MRI findings were not classic for either lesion, given the patient’s age and the CT findings, our initial assumption was that this lesion was a colloid cyst.

There are only two other reports of third ventricular dermoid cysts (Table 1). The first case occurred in a three-year-old female who had a history of nystagmus and headaches since birth. CT findings showed a mixed density, irregularly round mass with teeth within the cyst and associated obstructive hydrocephalus. Surgical excision was not performed, and hydrocephalus was managed with a ventriculoperitoneal shunt (VPS). Additionally, she had an associated dermal sinus tract that extended into the nasal septum [6]. The other reported case was in a nine-year-old female with one year of headaches and fever with two months of ataxic gait and blurred vision. CT findings demonstrated a hypodense, irregularly-shaped third ventricular mass with obstructive hydrocephalus. MRI revealed a fat-filled cyst with hair radiating from the center. Management was not described in this case [7].

**Table 1:** Reported third ventricular dermoid cysts

Author and year	Description	CT findings	MRI findings
Brydon (1992) [6]	Three-year-old female, history of nystagmus and headaches since birth	Cyst with teeth contained within the cyst, obstructive hydrocephalus	Not available
Iyer and Sanghvi (2008) [7]	Nine-year-old female, 1 year of headaches and fever, and 2 months of ataxic gait and blurred vision	Islands of fat and calcification, obstructive hydrocephalus	Fat filled cyst with hair radiating from the center
This case	64-year-old male presented with altered mental status	Hyperdense, spherical third ventricular, obstructive hydrocephalus	T1 hyperintense lesion without diffusion restriction, T2 hypointense

In comparison, our patient presented in his seventh decade and he had no report of progressive symptoms and had no evidence of a congenital anomaly or dermal sinus tract. The two previous cases had obvious dermoid cyst characteristics on imaging, such as hair or teeth. Imaging of our patient did not demonstrate such characteristics and more closely resembled a colloid cyst. Pertinently, in May 2008, our patient had a CT scan without any evidence of a third ventricular lesion, confirming that this was not a congenital lesion. This dermoid cyst may have developed from iatrogenically implanted ectodermally derived skin cells when the EVD was placed in July 2019. In Per et al.’s review of iatrogenically lumbar epidermoid cysts, the range between lumbar puncture and presentation was two to 23 years [13]. If this dermoid cyst was iatrogenic, our patient may have presented earlier due to the relatively small amount of space afforded to lesions at the foramen of Monro before causing obstructive hydrocephalus. Upon review of the imaging there was evidence of an underlying third ventricular mass on CT head only a month after EVD placement (Figure 1b). This could have been an indolent lesion obscured by hemorrhage that may have been identified on follow-up; however, the patient was unfortunately lost to follow-up.

## Conclusions

In conclusion, dermoid cysts are rare, benign intracranial lesions. Our patient’s third ventricular lesion was initially misdiagnosed as a colloid cyst based on imaging characteristics and location with biopsy proving the lesion to be a dermoid cyst instead. Third ventricular dermoid cysts are very rare lesions that vary significantly in both clinical presentation and management. Our case demonstrates the first-reported third ventricular dermoid cyst in an adult.
